# Cost-effectiveness analysis of molecular testing for cytologically indeterminate thyroid nodules

**DOI:** 10.1186/s40463-022-00604-7

**Published:** 2022-12-21

**Authors:** Navjit Dharampal, Kristine Smith, Adrian Harvey, Ralf Paschke, Luke Rudmik, Shamir Chandarana

**Affiliations:** 1 Section of Thoracic Surgery, Providence Cancer Institute, Portland, USA; 2 Department of Otolaryngology, University of Utah, Salt Lake City, USA; 3 Section of General Surgery, Department of Surgery, University of Calgary, Calgary, Canada; 4 Division of Endocrinology, University of Calgary, Calgary, Canada; 5 Section of Otolaryngology, Department of Surgery, University of Calgary, Foothills Medical Centre, North Tower Rm 1012, 1403 29 St NW, Calgary, AB T2N2T9 Canada; 6 Section of Surgical Oncology, Department of Oncology, University of Calgary, Calgary, Canada

**Keywords:** Cost-effectiveness analysis, Molecular testing, Afirma, Indeterminate thyroid nodule

## Abstract

**Background:**

Thyroid nodules affect up to 65% of the population. Although fine needle aspirate (FNA) cytology is the gold standard for diagnosis, 15–30% of results are indeterminate. Molecular testing may aid in the diagnosis of nodules and potentially reduce unnecessary surgery. However, these tests are associated with significant costs. The objective of this study was to evaluate the cost-effectiveness of Afirma, a commercially available molecular test, in cytologically indeterminate thyroid nodules.

**Methods:**

The base case was a solitary thyroid nodule with no additional high-risk features and an indeterminate FNA. Decision tree analysis was performed from the single payer perspective with a 1-year time horizon. Costing data were collected through micro-costing methodology. A probabilistic sensitivity analysis was performed. The primary outcome was the incremental cost effectiveness ratio (ICER) of cost per thyroid surgery avoided.

**Results:**

Over 1 year, mean cost estimates were $8176.28 with 0.58 effectiveness for the molecular testing strategy and $6016.83 with 0.07 effectiveness for current standard management. The ICER was $4234.22 per surgery avoided. At a willingness-to-pay (WTP) threshold of $5000 per surgery avoided, molecular testing is cost-effective with 63% certainty.

**Conclusion:**

This cost-effectiveness analysis suggests utilizing Afirma for indeterminate solitary thyroid nodules is a cost-effective strategy for avoiding unnecessary thyroid surgery. With a $5000 WTP threshold, molecular testing has a 63% chance of being the more cost-effective strategy. The cost effectiveness varies based on the cost of the molecular test and the value of Afirma for patients with indeterminate thyroid nodules depends on the WTP threshold to avoid unnecessary thyroid surgery.

**Graphical Abstract:**

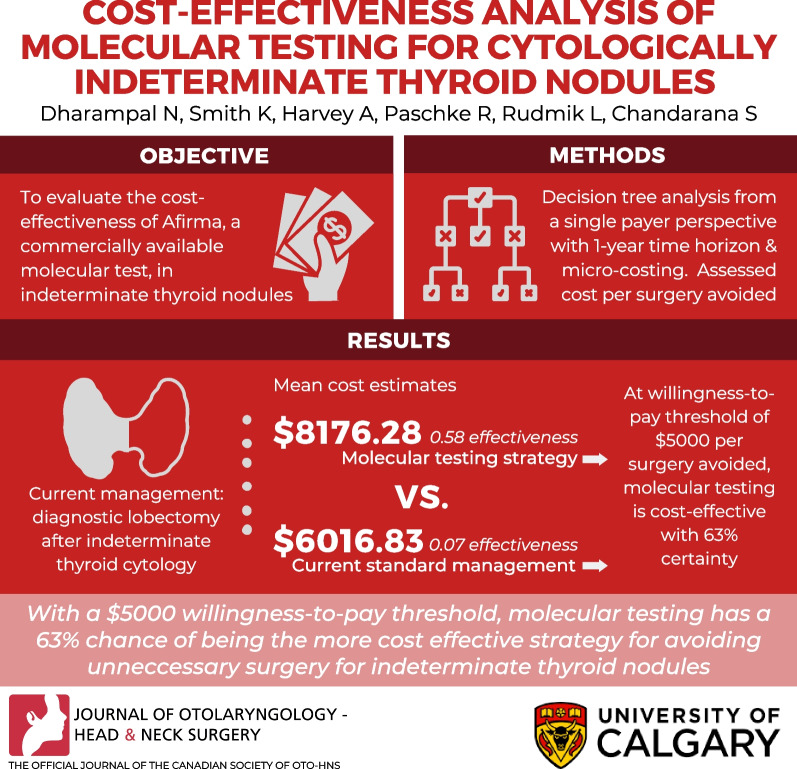

## Background

Thyroid nodules affect up to 65% of the general population and pose a significant diagnostic challenge [1]. Although fine needle aspirate cytology (FNAC) is the gold standard for stratifying risk of cancer within thyroid nodules, 15–30% of results are indeterminate [2–4]. The likelihood of malignancy in these indeterminate nodules varies significantly [3, 5]. Historically, a large proportion of these indeterminate nodules have been managed with a diagnostic lobectomy, which is effectively an excisional biopsy of the lesion. Ultimately, a majority of these nodules prove to be benign on final surgical histopathology [6]. Consequently, patients undergo unnecessary surgery, with exposure to perioperative risk and anxiety associated with the procedure. In addition, a small proportion of patients with normal pre-operative thyroid function will require lifelong thyroid hormone supplementation following surgery. There are also significant added direct costs to the single-payer Canadian healthcare system as well as indirect costs related to the recovery time for the patient and its impact on society, and this puts further strain on sparse resources such as access to operating room time and inpatient hospital beds.

Molecular testing is an innovative diagnostic tool which aids in risk stratification of cytologically indeterminate thyroid nodules [6, 7]. An example of a molecular test is Afirma – a gene expression classifier that assesses the expression of 142 genes [7]. The result of this test is then used in a proprietary algorithm to determine if a nodule is cytologically favored to be benign or malignant. However, the test itself is associated with significant cost, which is currently not covered by the Canadian healthcare system. Nodules categorized as indeterminate based on the Bethesda system (either Bethesda III or IV) are estimated to have a relatively low pre-test probability of cancer (5–15% and 15–30% respectively) [3]. As such, a molecular test with sufficient sensitivity may serve to “rule-out” cancer and allow patients to avoid surgery in favor of surveillance. Indeed, some authors have reported a significant decrease in the need for diagnostic lobectomy following introduction of this gene expression classifier [8].

The objective of this study was to evaluate the cost-effectiveness of using the Afirma test, within the context of a Canadian health care system, for cytologically indeterminate solitary thyroid nodules as compared to the current practice of diagnostic lobectomy. Cytologically indeterminate nodules included Bethesda III (atypia of unknown significance, AUS; follicular lesion of unknown significance, FLUS) and IV (follicular neoplasm, FN; suspicious for follicular neoplasm, SFN). We compared two strategies: (1) molecular testing with Afirma followed by diagnostic lobectomy as necessary; and (2) standard management (diagnostic lobectomy after indeterminate FNAC, no molecular testing). The results of study have the potential to inform policy makers on whether molecular diagnostic testing may alleviate some of the costs to the Canadian health care system, while concomitantly saving patients from unnecessary surgery.

## Methods

### Base case

**Fig. 1:**
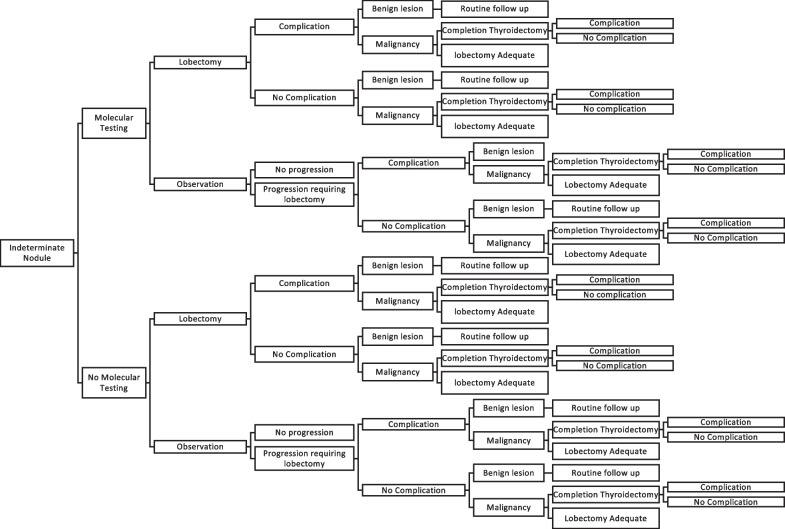
Decision tree model for indeterminate thyroid nodule

The base case (Table 1) utilized in this study was a patient with a solitary thyroid nodule between 1 and 4 cm in size, with no concerning features on physical exam and with indeterminate cytology (AUS/FLUS and FN/SFN) on FNA. The base case patient had no adverse risk factors and the ultrasound risk stratification was either low or intermediate risk, as per American Thyroid Association radiologic risk factors. The management strategies of the hypothetical patient were based on the 2015 American Thyroid Associated guidelines [6]. Decision tree analysis was performed from the payer perspective (government third party payer) with a 1-year time horizon. Two main approaches to an indeterminate thyroid nodule were modeled: (1) diagnostic lobectomy for a nodule with indeterminate cytology, and (2) use of a molecular test for nodules with indeterminate cytology, followed by surgery as necessary. The decision tree is presented in Fig. 1.

**Table 1:** Base case characteristics

Solitary thyroid nodule (1-4 cm)
Indeterminate cytology on FNA (AUS/FLUS or FN/SFN)
No patient risk factors (previous radiation, family history of thyroid cancer, genetic disorder)
Low or intermediate ultrasound features

*AUS* atypia of unknown significance, *FLUS* follicular lesion of unknown significance, *FN* follicular neoplasm, *SFN* suspicious for follicular neoplasm

### Molecular genetic test

The molecular test (Afirma Gene Expression Classifier, Veracyte, San Francisco, CA) assessed in this study evaluates cytologically indeterminate thyroid nodules by testing the mRNA expression level for 142 genes. The nodule is then classified as molecularly benign or suspicious for malignancy based on a proprietary algorithm. It has been noted to have a high negative predictive value of > 94%; thus, is it used as a “rule out” test for thyroid malignancy [6, 9, 10]. Accuracy rates of the test were based on validation studies of Afirma, using prevalence rates from a North American setting [11]. Nodules predicted to be benign do not require further assessment, only routine observation, whereas those favored to be suspicious should undergo diagnostic lobectomy.

### Risk, probability and utility estimation

Transition probabilities and utility values for health states utilized in the model were collected from published literature. Missing values were elicited by experts in surgery and endocrinology at the University of Calgary. Probability values are presented in Table 2 [8, 11–13].

**Table 2:** Probability values

Health State	Probability	References
*Afirma*		
Lobectomy	0.389	11
Malignancy diagnosed on lobectomy	0.440	11, EO
Eventual lobectomy following observation	0.400	13, EO
Malignancy in nodules being observed	0.091	11
*Current Practice*		
Lobectomy	0.740	8
Malignancy diagnosed on lobectomy	0.195	4, 5
Eventual lobectomy following observation	0.400	13, EO
Malignancy in nodules being observed	0.160	11, EO
*Both*		
Complication following lobectomy	0.135	4, 5, EO
Complication following completion thyroidectomy	0.117	4, 5, EO
Completion thyroidectomy following lobectomy for malignancy pathology	0.042	12

*EO* expert opinion

### Cost estimation

The costing perspective was the government payer, reflecting the single payer system used in Canada. Costs were collected through a micro-costing methodology, whereby monetized unit costs for each resource consumed during the surgical management of thyroid nodules were captured [14]. Resource unit costs were procured from Alberta Health Services (AHS) purchasing database, and by contacting AHS pharmacy, Calgary Lab Services, and the AHS diagnostic imaging department. Operating room costs were obtained by retrospective review of intraoperative nursing costing sheets for 5 index cases per procedure. Labor costs were defined using the provincial physician fee schedule for Alberta, Canada. Unit costs are summarized in Table 3. The frequency and costs incorporated in “postoperative complications” arms of our model included wound infection, transient or permanent hypocalcemia, recurrent nerve injury, chyle leak and post-operative hematoma. The costs were weighted by relative frequencies. The cost of the Afirma test was also based on current Canadian data. Discounting was not utilized due to the short cycle length (1 year).

**Table 3:** Costs

Item	Cost (CAD)
Molecular test (*Afirma)*	4938
Observation (for 1 year)	466
Lobectomy	4937
Complications associated with lobectomy	4087
Follow up for benign pathology following lobectomy	240
Follow up for malignant pathology following lobectomy	466
Completion total thyroidectomy	7269
Complications associated with completion thyroidectomy	4094

### Cost-effectiveness analysis

The decision tree model was run using the fixed base case parameters as listed in Table 1 with microsimulations of 1 million patients over a 1-year cycle. Mean costs and transition values for each management strategy were determined. The unit of effectiveness was the number of unnecessary surgeries avoided. Unnecessary surgery was defined as thyroid surgery that occurred where the final nodule pathology was benign. The primary outcome measure of this study was the incremental cost-effectiveness ratio (ICER) of cost per surgery avoided. The ICER was calculated as incremental cost (cost of strategy A–cost of strategy B) divided by incremental effectiveness (number of surgeries avoid using strategy A – number of surgeries avoid using strategy B). Model simulation was executed and analyzed with Tree Age Pro Suite 2017 (TreeAge Software, Inc., Williamstown, MA).

### Sensitivity analysis

We performed probabilistic sensitivity analyses with 10,000 Monte Carlo simulations to derive 95% uncertainty intervals. The willingness to pay (WTP) was set to $5000. One-way sensitivity analyses were performed on all probabilities and costs to interrogate the contribution of model variables.

## Results

### Cost-effectiveness analysis

Following model iterations representing 1-year of management of a cytologically indeterminate thyroid nodule, current practice of diagnostic lobectomy resulted in a mean cost of $6016.83 per patient and 0.07 mean effectiveness in avoiding unnecessary surgery. Use of the molecular testing to manage the indeterminate nodule resulted in a mean cost of $8176.28 and mean effectiveness of 0.58. Therefore, the use of the molecular test management strategy has a slightly higher cost (+ $2159.45) but with an improved effectiveness (+ 0.51). This resulted in an ICER of $4234.22 per unnecessary surgery avoided (ICER = [$8176.28—$6016.83]/[0.58 – 0.07]).

### Sensitivity analysis

In the one-way sensitivity analysis, the cost of the molecular test was the variable contributing most heavily to cost-utility in the model; the second contributor was the cost of a diagnostic lobectomy. Threshold analysis revealed the molecular testing management strategy became cost neutral at a test cost of $2778.06 and dominant with any cost lower than this value.

**Fig. 2:**
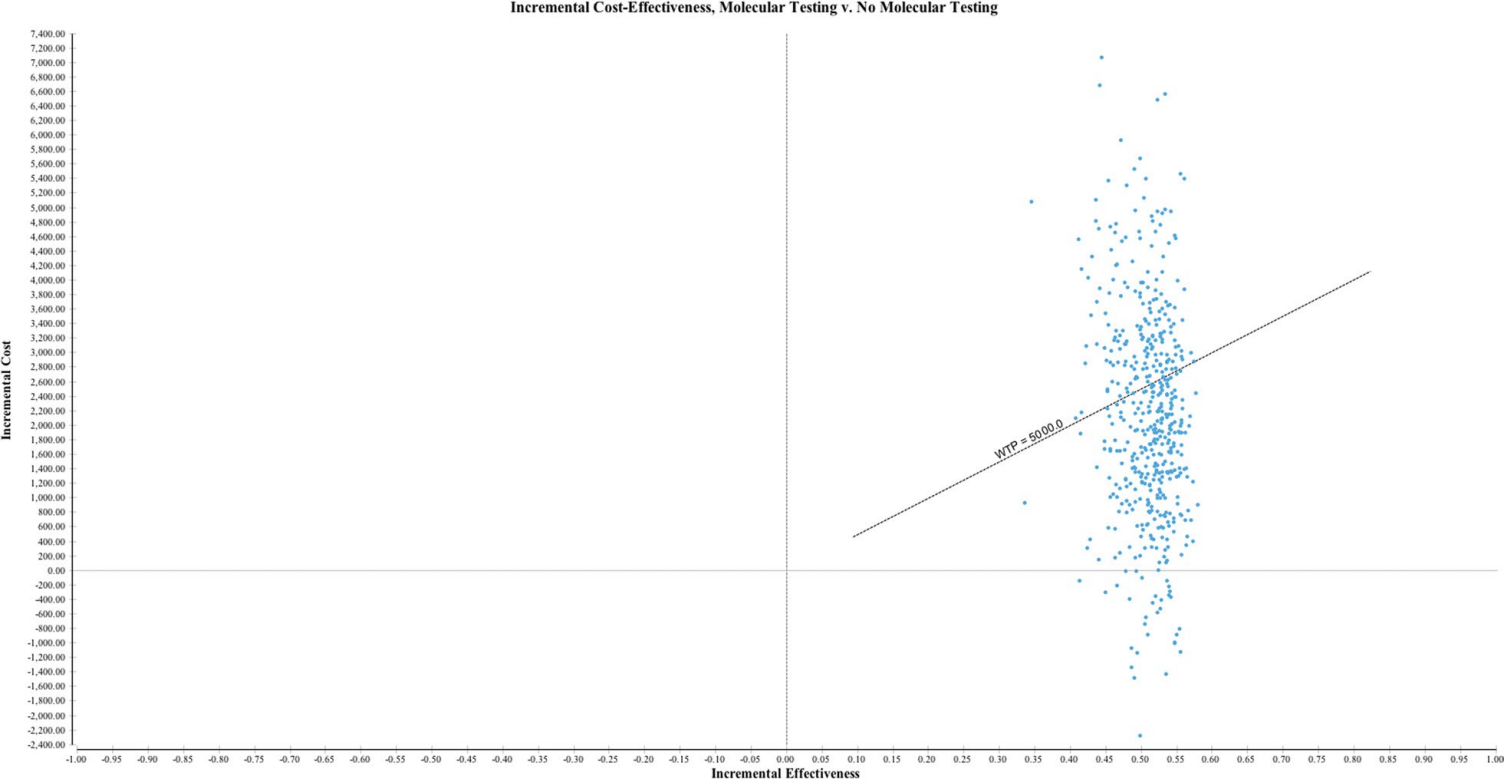
Probabilistic sensitivity analysis

Probabilistic sensitivity analysis is presented as a scatterplot in Fig. 2. With a willingness-to-pay set at $5000, the molecular testing strategy was the more cost-effective strategy with 62.6% certainty. Molecular testing was always more effective, as illustrated by no trials falling to the left of the scatterplot. Molecular testing was dominant in 6.4% trials.

## Discussion

This cost-effectiveness model and analysis suggest utilizing Afirma in the testing of indeterminate solitary thyroid nodules is a cost-effective strategy for avoiding unnecessary thyroid surgery. At a willingness-to-pay threshold of $5000 per surgery avoided, the Afirma strategy is the more cost-effective strategy with a certainty of 62.6%. Although including Afirma within the treatment algorithm has an increased cost compared to standard management, $8176.28 and $6016.83 respectively, molecular testing had a superior effectiveness in avoiding unnecessary surgeries (0.58 versus 0.07). Thus, the results of this study may help inform the decisions of clinicians and patients as they weigh the case-by-case usefulness of molecular testing in indeterminate thyroid nodules. For many patients and health care providers alike, this incremental increase in cost when using molecular testing (approximately $4000) is easily offset by the number of surgeries avoided and the resultant cost savings.

An interesting finding of this study is that cost effectiveness varies significantly based on the cost of the molecular test. In the one-way sensitivity analysis, the cost of the molecular test was the heaviest contributor to cost-utility. Interestingly, the test became cost-neutral at a cost $2778.06 and was dominant at lower costs (i.e. at a lower cost of the test, this strategy would be both cheaper and more effective). Therefore, as the costs of the molecular test decreases, testing becomes the clear choice in managing indeterminate solitary thyroid nodules. Other molecular tests are in development and are likely to be made available at lower costs [15]. Of note, while it has been shown that patients with a high pre-test probability of malignancy, based on worrisome findings on high-fidelity ultrasound with standardized reporting, are less likely to benefit from molecular testing, this study assumes the base case of an individual with low/intermediate risk features on ultrasound [16]. Further, high quality ultrasound with standardized reporting is not yet available in many Canadian regions, and therefore does not depict the current reality of these regions.

While this study suggests molecular testing is a cost-effective strategy, the results from previous studies have been varied [13, 17–19]. These conflicting results likely relate to the differences in model construction, outcome measures and cost estimates in different health systems. Despite this, only a few studies conclude molecular testing is not cost-effective and is, instead, dominated by standard practice.

A difference when comparing these studies to the current study is the choice of outcome measure. Some previously published studies assess effectiveness through Quality Adjusted Life Years (QALYs), as opposed to surgeries avoided, used in this study [17, 20]. QALYs gained is an established outcome measure in cost-effectiveness analyses, allowing for inclusion of more broad health states related to less quantifiable metrics such as the impact of time away from work, subsequent diagnostic testing, anxiety related to the disease state and the societal impact. While QALY is an important metric when assessing the cost-efficacy of two different *treatment interventions* (which have long-term consequences on patient outcome), it may not be the optimal metric for assessing the immediate impact of a *diagnostic test*. The utility of a diagnostic test such as Afirma is best evaluated by determining its impact on avoiding a more invasive diagnostic procedure (lobectomy). It has little impact on the long-term disease state for the individual patient. Regardless of choice of treatment strategy, patients in both arms will be subject to some degree of follow up testing, and the anxiety related to this. Furthermore, given that overall outcomes for patients diagnosed with indeterminate thyroid nodules is excellent, and the risk of surgical complications that negatively affect long-term quality of life is low, the broad health states captured by QALY are not dissimilar, regardless of whether a molecular test was used [18]. Using QALY would therefore significantly dilute the impact of the cost-efficacy of a molecular test. Finally, the health utility values utilized in the analyses using QALYs are extrapolated from a small sample survey study and may not be accurate, and attaching costs to these utilities is even more problematic [21]. This is in large part due to the fact that factors that affect global heath states (time away from work, anxiety) occur inconsistently between individual patients, and the degree to which they occur varies immensely. This is particularly important as models were highly sensitive to the valuation of health states and, hence, inaccurate health utility values would challenge the robustness of the model [17]. Using *surgeries avoided* as an outcome of effectiveness more directly addresses the strength of using a molecular test and provides a more practical and immediate sense of the benefit gained. Similar to our study, a study assessing surgeries avoided as an outcome of effectiveness found molecular testing to be more effective compared to standard practice [18]. Additionally, a study assessing the cost-effectiveness of molecular testing compared to diagnostic thyroid lobectomy using correct diagnosis as the outcome, rather than QALY, found molecular testing to be the superior, more cost-effective strategy [24].

When constructing the decision tree model, another variable that differs across studies is extent of ongoing surveillance required for indeterminate thyroid nodules deemed benign or “negative” by molecular testing. Models concluding standard practice to be more cost-effective compared to molecular testing included ongoing follow up and surveillance of “negative” nodules over the course of the model, typically 5 years [17, 20]. The appropriate surveillance for these “negative” nodules has yet to be elucidated and annual follow up may be excessive and therefore incur unnecessary costs, leading to an inflated cost estimation of the molecular testing strategy. Further, regardless of use of a molecular test, the long-term outcomes of the two treatment arms are not dissimilar in terms of favourable clinical outcomes for indeterminate thyroid nodules, as well as the use of ongoing tests for surveillance. To avoid masking the immediate impact of the molecular test on avoiding surgery, our model’s time horizon included a surveillance duration of one year. During this one-year time horizon, standardized follow-up testing and the associated probabilities of negative outcomes were incorporated into the observation portion of the model, to allow for real-world simulation. The creation of a robust costing model with a finite time horizon allows for direct comparison of several commercially available molecular tests, and will highlight any subtle differences in cost-efficacy.

It is important to note that nodules with cytology consistent with either Bethesda III and IV were pooled into one analysis. While in theory, a separate analysis for each category could yield a separate cost-efficacy outcome, there were several practical reasons to amalgamate these two categories. Firstly, the ATA guidelines state that both Bethesda III and IV nodules are deemed indeterminate in terms of malignancy risk and could be managed via diagnostic lobectomy. The reported range of malignancy for both categories is wide and overlapping; in Table 1 of the ATA guideline [4], the range for Bethesda III is 6–48% and Bethesda IV is 14–34%. Therefore, there would be little value in running the model separately for Bethesda III and Bethesda IV, given the probability of malignancy of the two categories are similar. Additionally, both Bethesda III and IV nodules are suitable candidates for molecular testing. Finally, with regard to model construction, while the model could stratify for Bethesda risk category in the standard treatment arm, the rates of malignancy in the Afirma arm, stratified by Bethesda category, are not well known. This would force the model to pool the analysis in one treatment arm, while not pooling in the other. To avoid this, a decision was made to remain consistent with previously published studies that have similarly attributed a pooled malignancy risk to both Bethesda III and IV nodules [4, 5]. In this study, that risk was 19.5%. It should be noted that the model did vary the malignancy risk to an upward limit of 50% (to accommodate for centres with higher rates), but this did not have a significant impact on the outcome of the analysis.

The ATA Guideline recommendation 15 suggests that nodules with initial cytology of AUS/FLUS cytology could undergo either repeat FNA *or* molecular testing [4]. Our model therefore did not consider repeat FNA as an option since the goal was to address the impact of a decision to use molecular testing instead. Further given that repeat FNA is inconsistently used in practice, eliminating this option from the model allows for evaluation of a homogeneous patient population that was subjected to the same preliminary investigations.

There are several unique strengths of our study. Firstly, this is the first cost-effectiveness analysis for Afirma in the management of indeterminate nodules using Canadian specific data and a single payer model. This is particularly important as the cost of the test varies among countries. The results of this study are contextualized to the Canadian health care system, can provide unique insight into the value of molecular testing in Canada, and inform potential decisions to fund this test by provincial health systems. Secondly, the costs employed in this model are not reported or aggregate costs. Instead a more accurate micro-costing approach was used to populate our model to allow for a more robust and accurate cost estimation [14, 22]. Thirdly, we used a short time horizon for this model: one year. While some studies use a longer time horizon, these models may dilute the impact of a diagnostic test as the contribution to cost or efficacy is primarily in the first cycle or year of the model. The further iterations become more reliant on factors that are difficult to control (and not related to the molecular test), such as final pathology, risk of recurrence, and relevant findings on ongoing surveillance investigations, which may trigger further costly interventions. Fourthly, while Afirma was the molecular test used to construct the costing model for this study, the model now allows for the substitution of any commercially available molecular test, to allow for comparisons to be made. Lastly, the treatment algorithm utilized in the model is based on the most recent ATA Guidelines, published in 2015, thereby making the model more aligned with current clinical practice and the most up-to-date cost-effectiveness analysis of molecular testing.

There are some limitations with this study. Firstly, all models inherently must incorporate assumptions and expert opinion must be used where there is a paucity of published literature. In this model, all nodules that grew in size following a negative Afirma test underwent diagnostic lobectomy. However, the rates of growth in this population are unknown, as is the rate of eventual malignancy. Therefore, identical rates of growth for Afirma-negative nodules were used as for all thyroid nodules which have not undergone testing. This may overestimate rate of growth in the Afirma-negative nodules, and therefore overestimate the probability of diagnostic lobectomy, malignancy, complications, and the related costs [19]. However, this may strengthen the conclusion that Afirma is cost-effective, as over-estimates of the cost associated with the Afirma strategy would bias the results towards standard practice as the more effective approach. Secondly, the willingness-to-pay threshold varies from previously published literature. The most common value cited is a WTP of $100,000/QALY gained. However, as the primary outcome in this paper is “unnecessary surgery avoided”, the WTP threshold is not related to QALYs and rather surgery avoided. To provide the most conservative estimate possible, a WTP threshold similar to the cost of surgery ($5000) was used, on the assumption that an individual would be willing to pay at least that same amount for the test, in order to avoid the surgery. Of note, costs associated with surgery are already accounted for in the costing model and the effectiveness metric measures only the willingness to pay over and above the financial cost of surgery. Practically, a payer would likely be willing to pay much more than $5000, given that this cost does not account for the monetary loss of time away from work, and decreased productivity in relation to contribution to society. Similar approaches have been used in other papers where the long-term quality of life does not differ substantially between the two groups, as is the case in this patient population. Additionally, other published WTPs for surgery avoided are much higher. For example, a study comparing early versus late tracheostomy cited a WTP of $80,000 per tracheostomy avoided [23]. Had a larger WTP threshold been used, the certainty that molecular testing was the more cost-effective strategy would have increased, however the goal was to be conservative in the conclusions. Thirdly, we utilized a healthcare perspective (single government payer) and thus this model does not include societal costs such as time away from work, income loss, and delays for procedures. These are important yet difficult factors to incorporate. In addition, we utilized the micro-costing approach based on local data, making our data more relevant to the Canadian context, but limiting the generalizability of our findings to other settings outside of Canada. Despite these limitations, the unique strengths and perspective of this study support the conclusion of this cost-effectiveness analysis.

## Conclusion

For indeterminate thyroid nodules, the results of our economic model suggest molecular testing is a superior strategy compared to standard management in decreasing the number of unnecessary surgeries (i.e. surgery performed for ultimately benign disease). The incremental cost is $4234.22CAD per unnecessary surgery avoided. The model was most sensitive to the cost of the test itself. As such, as the cost of molecular testing decreases, the cost-effectiveness of the test increases. These results may help health policy makers and resource managers make informed decisions regarding the value of molecular testing in the management of indeterminate thyroid nodules. Specifically, although the upfront cost of the molecular test may appear high, its use avoids unnecessary (and even more costly) surgery. This study also provides a robust cost-efficacy model to which additional emerging molecular tests can be applied to allow for comparison across tests.

## Data Availability

All data generated or analyzed during this study are included in this published article.
